# Vaccinations Included in the National Immunization Calendar as a Tool to Tackle Antimicrobial Resistance: Current Evidence for Selected Pathogens in Italy

**DOI:** 10.3390/vaccines13111141

**Published:** 2025-11-05

**Authors:** Giulia Carla Marchetti, Paolo Giuseppino Castiglia, Andrea Lombardi, Federico Marchetti, Giovanni Gabutti

**Affiliations:** 1 Clinic of Infectious and Tropical Diseases, Department of Health Sciences, University of Milan, ASST Santi Paolo e Carlo, 20142 Milan, Italy; giulia.marchetti@unimi.it; 2 Department of Medicine, Surgery and Pharmacy, University of Sassari, Italian Scientific Society of Hygiene, Preventive Medicine and Public Health (SItI), 07100 Sassari, Italy; castigli@uniss.it; 3 Infectious Diseases Unit, Fondazione IRCCS Ca’ Granda Ospedale Maggiore Policlinico, Department of Pathophysiology and Transplantation, University of Milan, 20122 Milan, Italy; andrea.lombardi@unimi.it; 4 GSK Vaccines, 37135 Verona, Italy; federico.e.marchetti@gsk.com; 5 Italian Scientific Society of Hygiene, Preventive Medicine and Public Health (SItI), Cogorno (Ge), Adult Immunization Board (AIB), 16030 Cogorno, Italy

**Keywords:** antimicrobial resistance, antibiotic resistance, vaccines, review, national immunization plan, Italy

## Abstract

**Background/Objectives**: Antimicrobial (AMR) and antibiotic resistance (AR) remain major growing issues around the world. According to WHO, vaccinations play a strategic role in tackling AMR/AR; the new (upcoming) or existing vaccines (both viral and bacterial) directed toward resistant pathogens, may consistently reduce the overall burden of infectious diseases across the population. The objective of the present work is to review the available evidence on the impact that immunization schedules might exert in terms of antibiotic use reduction, focusing on vaccinations included in the Italian National Immunization Plan (NIP). **Methods**: A targeted literature search, limited to 2015–2025, was performed in the PubMed database to identify the available evidence on the impact that vaccinations exert on antibiotic use or reduction. **Results**: The search provided evidence on the potential impact that immunizations included in the NIP might exert in tackling AMR/AR. Influenza and pneumococcal vaccinations proved to be those with the broadest base of evidence in reducing antibiotic prescriptions. Preliminary local evidence also suggests an impact on reducing antibiotic use for RSV immunization among adults and older adults. Rotavirus vaccination proved to reduce antibiotic prescriptions, while varicella disease was associated with a relevant use of antibiotics. **Conclusions**: Vaccines are essential in the fight against AMR/AR. In this review the evidence on the impact that vaccinations included in the NIP may exert was compacted. The impact of vaccines on reducing AMR/AR should be recognized by Italian stakeholders and strategies and implementation plans should always include vaccines as interventions to reduce AMR/AR.

## 1. Introduction

### 1.1. The General Frame of Antimicrobial Resistance

Antimicrobial resistance (AMR) is a global health crisis that threatens the effectiveness of treatments for infections caused by bacteria, viruses, fungi, and parasites. The World Health Organization (WHO) reported that AMR directly caused 1.27 million deaths worldwide in 2019 [1]. Misuse of antimicrobial drugs in human healthcare, veterinary, and agricultural settings is the main driver of multidrug-resistant pathogens, compounded by challenges in developing new antibiotics and slow progress in alternative technologies [2,3]. AMR disproportionately impacts low- and middle-income countries, exacerbating inequalities and imposing significant economic costs, with healthcare expenses projected to increase by one trillion dollars by 2050 [4,5]. During the 2024 European Antibiotic Awareness Day, it was revealed that over 35,000 people in the EU die annually from antimicrobial-resistant infections, with mortality rates comparable to influenza, tuberculosis, and HIV/AIDS combined [6]. Additionally, 4.3 million patients in the EU acquire healthcare-associated infections each year, many of which are resistant to antimicrobials, limiting treatment options [6].

WHO has identified key actions to combat AMR, including infection prevention, robust surveillance, access to quality diagnostics and treatments, and fostering innovation [7]. To improve the judicious use of anti-bacterials, WHO classified antibiotics into three categories: Access (low resistance risk, targeted for 70% of global use by 2030), Watch (broad-spectrum antibiotics for severe infections), and Reserve (last-resort treatments for multidrug-resistant infections). Excessive antibiotic use, particularly in the Watch category, remains a significant issue, highlighting the need for stewardship programs and equitable access to antibiotics [8].

In 2015, WHO launched the Global Action Plan on Antimicrobial Resistance, focusing in raising awareness through education, improving surveillance and research, reducing infections via sanitation and hygiene, optimizing antimicrobial use in humans and animals, and increasing investment in new drugs, diagnostics, and vaccines [9].

**Figure 1:**
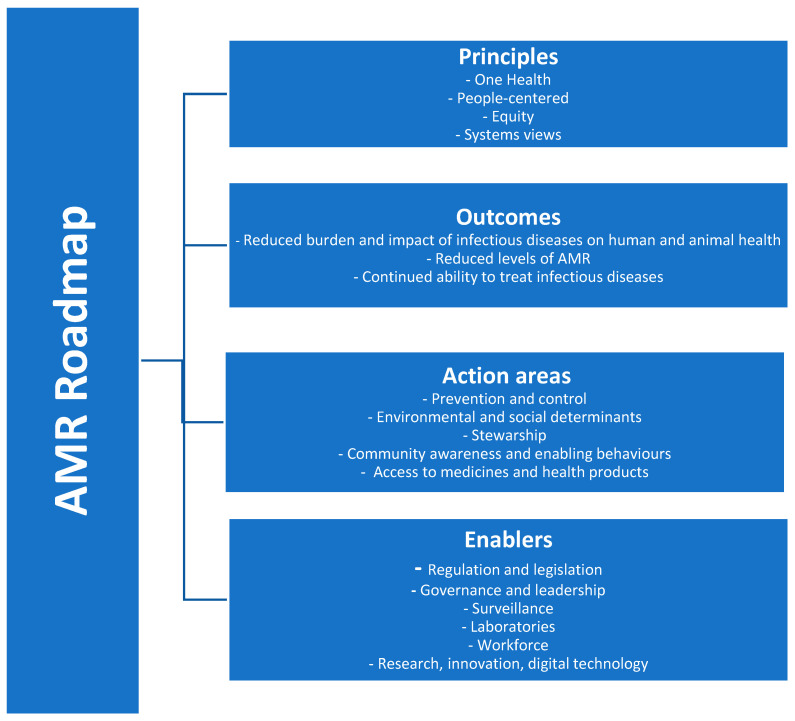
Roadmap on antimicrobial resistance for the WHO European Region 2023–2030 (modified from [14]).

In 2021, WHO released an Action Framework emphasizing vaccines’ role in preventing infections and reducing antimicrobial use, with three goals: maximizing vaccine impact on AMR, updating vaccine-related guidance, and improving awareness through education and communication [10]. In 2023, WHO developed the “People-centered approach to addressing antimicrobial resistance” campaign, focusing on overcoming barriers to accessing health services for preventing, diagnosing, and treating drug-resistant infections [11]. The One Health approach was defined by WHO as an integrated approach that aims to sustainably balance and optimize the health of people, animals, and ecosystems [12]. By promoting interdisciplinary collaboration, One Health connects knowledge, data, and expertise from different disciplines and sectors and aims to overcome regulatory barriers, recognizing the connection between the health of humans, animals, plants, and the environment [12]. The Tracking Antimicrobial Resistance Country Self-Assessment Survey (TrACSS) system was developed to monitor AMR globally, encompassing human health, animal health, food, agriculture, and environmental factors, helping identify gaps and priorities for the updated Global Action Plan in 2026 [13]. At the European level, WHO introduced the AMR Roadmap 2023–2030 to guide countries in implementing and monitoring interventions to combat AMR [14]. The roadmap outlines five key areas and six enabling factors, offering adaptable, evidence-based strategies to improve human and animal safety against treatment-resistant infections by 2030 [14] (Figure 1).

### 1.2. The Italian Landscape

Antibiotic resistance (AR) in Italy is a serious health emergency, making the country one of the highest in Europe for infections and deaths caused by resistant bacteria [15].

Estimates vary, but according to AIFA (Italian Medicines Agency), AMR causes about 12,000 deaths per year, while other sources indicate a similar number (about 11,000). In 2023, Italy reported significant resistance to major antibiotic classes among monitored pathogens. The National Antimicrobial Resistance Surveillance System (AR-ISS) collects annual antibiotic susceptibility data for key pathogens (e.g., *S. aureus*, *E. coli*, *K. pneumoniae*) through hospital microbiology labs [16]. AR-ISS contributes to the European Antimicrobial Resistance Surveillance Network (EARS-Net) and, since 2020, to the global GLASS system coordinated by WHO [16]. Among the most worrying multi-resistant bacteria in Italy are *Acinetobacter* spp., *Enterococcus faecium*, and *Staphylococcus aureus* (MRSA) [16]. Italy faces high resistance rates compared to other European countries, especially in the North. As an example, in 2022, the percentage of resistance of *K. pneumoniae* to fluoroquinolones was 48.7% in Italy, compared to less than 15% in northern Europe [16]. The 2025 AIFA report on 2023 antibiotic use revealed increased consumption across age groups and in hospitals [15]. The Drug Resistance Index, which combines antibiotic use and resistance, rose in most regions for pathogens like *E. coli*, *S. pneumoniae*, and *E. faecium.* Overall antibiotic consumption increased, reaching 22.4 daily doses per 1000 inhabitants for systemic antibiotics and 28 daily doses per 1000 inhabitants for non-systemic antibiotics [15]. The economic impact of AR on Italy is significant. According to AIFA, antibiotic resistance costs the National Health Service about 2.4 billion euros per year; between 2022 and 2023, the consumption of antibiotics for systemic use increased by 5.4%, contradicting the goal of a 5% reduction by 2025 set by the National Plan to Combat AMR [15,17]. The Italian National Plan to Combat Antibiotic Resistance (PNCAR) 2022–2025 outlines strategic guidelines to address AR in human, animal, and environmental sectors. Key interventions include integrated surveillance of AR, antibiotic use, healthcare-associated infections (HAIs), and environmental monitoring; prevention of HAIs and infections in general by vaccination and zoonoses; and promoting appropriate antibiotic use and proper disposal of antibiotics and contaminated materials [17]. In parallel, the National Vaccination Plan 2023–2025 (PNPV) aims to reduce vaccine-preventable diseases through uniform strategies, including maintaining Polio-free status, eliminating measles and rubella, preventing HPV-related diseases, and achieving vaccination coverage targets. It also focuses on reducing vaccination inequalities, improving surveillance, digitizing vaccination registries, and promoting vaccination awareness among healthcare professionals. The PNPV highlights the role of vaccines in combating AR by preventing bacterial and viral infections, reducing antibiotic misuse, and supporting the development of vaccines against resistant pathogens [18]. Furthermore, in 2022, a number of Italian Scientific Societies endorsed the document “Recommendations for an effective strategy against antimicrobial resistance,” emphasizing vaccine prevention, innovation in antibiotic development, and appropriate antibiotic use as key tools to combat AMR [19].

### 1.3. The Role of Vaccines in Combating AMR

Vaccines are highly effective in preventing diseases that might otherwise require the use of antibiotics and/or antimicrobial agents to treat their symptoms and associated complications. Vaccines provide both individual protection and reduce the severity of infection by triggering herd immunity if high coverage rates are achieved and maintained. All this contributes to reducing both the incidence of infections (caused by both susceptible and resistant germs) and complications (possibly related to secondary infections), resulting in a reduction in antibiotic use.

The impact of vaccines on bacterial AMR correlates with a reduction in individual infection risk in vaccinated individuals, a prevention of transmission, a reduction in bacterial carriage, as well as of the pathogen population in the host, a reduction in the incidence of vaccine-preventable diseases, and a reduction in the need for care/treatment and therefore potential exposure to resistant pathogens [20,21]. This also reduces the possibility of pathogens acquiring resistance, reduces the individual risk and the transmission of resistant pathogens, and prolongs the effectiveness of available antibiotics [22].

The impact of viral vaccines on AMR is less intuitive, as antibiotic therapy is not indicated for viral infections. In this case, the mechanisms involved are the prevention of infections that often result in secondary bacterial infections and their associated antibiotic prescriptions, and the prevention of viral respiratory tract infections and their associated inappropriate antibiotic prescriptions [2].

In 2024, WHO published the document “Estimating the impact of vaccines in reducing antimicrobial resistance and antibiotic use” highlighting how existing vaccines could prevent up to 106,000 deaths, 9.1 million disability-adjusted life years (DALYs), $861 million in hospital costs, and $5.9 billion in productivity losses annually, all associated with antimicrobial resistance. These vaccines could also reduce antibiotic use by 142 million defined daily doses (DDDs) annually. Vaccines are therefore crucial in the fight against AMR and must be integrated into national and global AMR mitigation strategies and decision-making processes related to vaccine development, introduction, and use [23].

It would also be desirable to develop new vaccines against pathogens with high clinical impact and potential susceptibility to resistance or multidrug resistance. In this regard, there are some operational limitations to the development of new vaccines related to the poor understanding of immune evasion mechanisms, changing epidemiology, the difficulty of designing adequate clinical trials, and the lack and/or inadequacy of regulatory frameworks [22,24,25].

Regarding the medical costs associated with antimicrobial resistance (AMR) that are prevented by vaccines, there is limited evidence available. Only two modeling studies have provided estimates of the AMR-related costs avoided through pneumococcal vaccination, along with a few cost-effectiveness studies that primarily examined the impact of serotype replacement on the overall cost-effectiveness of vaccines. However, no cost-effectiveness studies have directly assessed the role of vaccines in slowing the progression of AMR. Additional research on the economic value and cost-effectiveness of vaccines in combating AMR would be instrumental in guiding resource allocation decisions and shaping development priorities [26].

The objective of the present work is to review the available evidence on the impact that immunization schedules might exert in terms of antibiotic use reduction, focusing on the vaccinations included in the Italian PNPV.

## 2. Methods

### Search Strategy

A targeted literature search was performed in June 2025 in the PubMed database to identify the available evidence on the impact that vaccinations exert on antibiotic use or reduction. The search strategy main string comprised the following:

Vaccinations antimicrobial resistance antibiotic use reduction (((“vaccin” [Supplementary Concept] OR “vaccin” [All Fields] OR “vaccination” [MeSH Terms] OR “vaccination” [All Fields] OR “vaccinable” [All Fields] OR “vaccinal” [All Fields] OR “vaccinate” [All Fields] OR “vaccinated” [All Fields] OR “vaccinates” [All Fields] OR “vaccinating” [All Fields] OR “vaccinations” [All Fields] OR “vaccination s” [All Fields] OR “vaccinator” [All Fields] OR “vaccinators” [All Fields] OR “vaccine s” [All Fields] OR “vaccined” [All Fields] OR “vaccines” [Supplementary Concept] OR “vaccines” [All Fields] OR “vaccine” [All Fields] OR “vaccines” [MeSH Terms] OR “vaccines” [All Fields]) AND (“drug resistance, microbial” [MeSH Terms] OR (“drug” [All Fields] AND “resistance” [All Fields] AND “microbial” [All Fields]) OR “microbial drug resistance” [All Fields] OR (“antimicrobial” [All Fields] AND “resistance” [All Fields]) OR “antimicrobial resistance” [All Fields]) AND (“antibacterial agents” [Pharmacological Action] OR “antibacterial agents” [Supplementary Concept] OR “antibacterial agents” [All Fields] OR “antibiotic” [All Fields] OR “antibacterial agents” [MeSH Terms] OR (“antibacterial” [All Fields] AND “agents” [All Fields]) OR “antibiotics” [All Fields] OR “antibiotic s” [All Fields] OR “antibiotical” [All Fields]) AND (“reduction” [All Fields] OR “reductions” [All Fields])).

The search was restricted to 2015–2025 to focus on the topic using newer methodology, and because in 2015 WHO released its plan regarding the use of vaccines to tackle AMR.

All results were screened, and papers dealing with vaccines and AMR as a general topic, or with pertussis, meningococcus ACWY, meningococcus B, pneumococcus, rotavirus, measles, mumps, rubella, varicella, respiratory syncytial virus (RSV), herpes zoster, and influenza were selected.

*Haemophilus influenzae* type B (Hib) vaccination was excluded from the analysis, as the Hib disease rate is very limited in Italy and *H. influenzae* invasive disease cases are caused by non-typable strains not prevented by the Hib vaccine [10,18].

Respiratory syncytial virus vaccination, either for pregnant women, newborns (with monoclonal antibodies), or for adults with or without risk conditions has not yet been introduced in the PNPV (at the time of writing); however, RSV immunization was recommended for older adults by the Board of the “Calendar for life”, an association of scientific societies [27], and was thus introduced in the search.

Despite being recommended in Italy, COVID-19 vaccination was excluded from the analysis, as data on the use, increase, or decrease in antibiotics to manage SARS-CoV-2 infections are still not clear.

No geographical or language restrictions were applied in the literature search. An integrative search for documents of interest was implemented on the web.

## 3. Results

The initial literature search yielded 270 papers, which were reduced to 176 after limiting the search to 2015 at the earliest. Focusing on the PNPV vaccinations, as in the objectives of the present work, papers were selected and summarized as follows. No papers dealing with the impact of vaccination on antibiotic use for the meningococcus B, measles, mumps, rubella, or herpes zoster vaccinations came up within the search.

### 3.1. Influenza Virus

Influenza viruses play a serious role in public health through annual epidemics, especially in at-risk populations such as the elderly, immunocompromised individuals, and people with chronic medical conditions [28]. The 2024 WHO report estimated that approximately 1 billion cases occur each year, of which 3–5 million are severe cases resulting in 290,000 to 650,000 respiratory-related deaths [23]. Influenza virus infection is often followed by inappropriate antibiotic prescription, as one of its main complications is community-acquired pneumonia (CAP), which typically requires antibiotic therapy due to secondary bacterial infection [28]. To limit influenza’s spread and complications, vaccination of high-risk groups as well as immunization of younger age cohorts is essential. In this regard, a randomized trial by Loeb et al. demonstrated that vaccinating children and adolescents with inactivated influenza vaccine significantly protected unimmunized members of rural communities against influenza [29]. Moreover, an interesting finding concerns the impact on antibiotic prescriptions, as vaccination was associated with a significant reduction in antimicrobial prescriptions (HR 0.58, 95% CI 0.34–0.99), indicating that influenza immunization can contribute to reducing unnecessary antibiotic use. These results were later confirmed in a follow-up trial by Wang et al., reporting a significant reduction in antimicrobial prescriptions (OR 0.69, 95% CI 0.49–0.98) through three different influenza seasons [30]. This hypothesis is in line with a recent Italian ecological study that analyzed the relationship between influenza vaccination coverage and AMR proportions over a two-year period. Considering a vaccination coverage of 64% among individuals over 64 years of age, the authors found a negative correlation with AMR rates in *K. pneumoniae* and *E. coli* (*p* < 0.001), particularly for *E. coli* resistant to fluoroquinolones. Similar evidence emerged for *P. aeruginosa* resistant to piperacillin/tazobactam [31].

Although the authors acknowledge that multiple mechanisms may underlie this association, the study highlights the potential value of influenza vaccination in reducing AMR rates among clinically relevant pathogens. Influenza vaccination findings are summarized in a systematic review in which pooled data from several RCTs showed a reduction in antibiotic prescriptions among infants and children aged 6 months to 14 years, as well as in adults aged 18 to 64 years [32], and in a subsequent systematic review and metanalysis [33].

### 3.2. Pneumococcus

*Streptococcus pneumoniae* represents a leading cause of pneumonia, meningitis, sinusitis, and acute otitis media. In the context of lower respiratory tract infections, *S. pneumoniae* is responsible for a considerable number of deaths, with up to 72% attributable to antimicrobial resistance [23]. The introduction of pneumococcal conjugate vaccines (PCV) marked a major advance, preventing severe infections and reducing antibiotic use, as highlighted by a randomized trial in Israel among daycare-attending toddlers, where use of the 9-valent PCV led to a significant drop in antibiotic consumption [34], playing a relevant role in tackling AMR.

These findings are consistent with a systematic review by Buckley et al., which reported moderate-certainty evidence that pneumococcal conjugate vaccines are associated with a modest reduction in rates of antibiotic purchases or prescriptions in children and infants across several randomized trials [32]; a recent metanalysis confirmed such figures [33].

Further evidence comes from a U.S. study that analyzed all cases of invasive pneumococcal disease between 1996 and 2004. The findings showed that immediately after the introduction of PCV7 there was a marked decrease in infections caused by penicillin-resistant strains. This effect was observed both in children under two years of age and in adults over 65, with reductions of 81% and 49%, respectively. Notably, the decline was also evident in unvaccinated populations, underscoring the importance of vaccination as a valuable tool against AMR [35].

Broadening the perspective, *S. pneumoniae* is also a leading pathogen for which vaccination may play a significant role in preventing AMR-related deaths and disability on a global scale. A recent modeling study by Kim et al. highlights how pneumococcal vaccination averted around 44,000 deaths and 3.8 million disability-adjusted life years (DALYs) associated with AMR in 2019. Moreover, the development of improved vaccines with broader efficacy could potentially double the number of avoidable AMR-related deaths in the future [36].

### 3.3. Varicella

Varicella (chickenpox) infections may cause secondary bacterial skin infections that require antibiotics. Although indirect, reductions in varicella incidence through vaccination curtails related antibiotic use substantially. An analysis was conducted by experts from Public Health England on 61,024 hospitalizations for chickenpox that occurred in the period of 2004–2017 in England in the absence of vaccination. In total, 8.1% of these hospitalizations developed a complication, with bacterial skin infections (11.25%) and pneumonia (4.82%) being the most frequent; moreover, the highest percentage of some complications such as encephalitis, meningitis, and pneumonia was observed in older children and adults [37]. More recently, a retrospective observational study was carried out in the time frame of 2016–2019 in Belgium using data from a longitudinal patient database in a primary care setting [38]. This study highlighted the substantial impact of chickenpox in Belgium, with a high rate of complications and antibiotic prescriptions. Indeed, 27.3% of children with chickenpox (3847 subjects; mean age 8.4 years) received a prescription for both systemic and topical antibiotics, and 2.7% received antivirals.

The highest antibiotic prescription rate (63.5%) was observed among subjects with complications and children aged < 1 year (63.5% and 41.8%, respectively) [38]. A model assessed the impact of chickenpox in the US on individuals under 18 years of age if unvaccinated. It was estimated that this scenario would result in over 500,000 chickenpox cases per year, with antiviral or antibiotic prescriptions being required in at least 23.9% of cases, mostly among unimmunized individuals. Estimates for scenarios with increasing vaccination coverage rates showed a significant reduction in costs resulting from antiviral and antibiotic prescriptions [39]. In Italy, mandatory vaccination for varicella was introduced in 2017 (associated with the measles–rubella–mumps vaccine: MMR). However, some regions implemented universal varicella vaccination (UVV) before 2017 and achieved a huge reduction in incidence rate (IR; 38.3 vs. 0.8/1000 person-years in 2017 and 2022, respectively). Regions that started UVV in 2017 registered a decrease in IR from 49.8/1000 person-years in 2017 to 3.2 in 2022 as well [40].

### 3.4. Respiratory Syncytial Virus (RSV)

In high-income countries (HICs), preventing RSV in infants has been associated with measurable decreases in antibiotic use, accounting in a study for 20.2% of all antimicrobial prescriptions in this population [41].

RSV infection leads to significant antibiotic prescriptions and associated costs, also among older adults in HICs. A study from the United Kingdom linked about 2.1% of all outpatient antibiotic prescriptions to RSV infection, especially in individuals over 75 years old [42]. An Italian study documented that RSV infection leading to hospitalization in a cohort of 289 enrolled patients with an RSV diagnosis over 60 years was associated with 15% higher healthcare costs compared to other causes of hospitalization [43]. A further analysis revealed that 181/289 (62.6%) patients received antibiotics systemically (ATC code J01) during the 1-year follow-up. Patients were prescribed with levofloxacin/ciprofloxacin (33.2%), amoxicillin/beta-lactamase inhibitor (24.6%), azithromycin/clarithromycin (24.6%), ceftriaxone/cefixime/cefditoren (23.2%), fosfomycin (8.0%) and other antibiotic classes (12.1%). Among these antibiotic groups, only amoxicillin/beta-lactamase inhibitor was included in the World Health Organisation (WHO)/AIFA AWaRe framework “Access” class, with the other prescribed antibiotics belonging to the “Watch” or “Reserve” classes [8,44]. The United Kingdom RSV immunization program, targeting individuals over 75 years and pregnant women, is projected to prevent 70,000 infant and 60,000 adult RSV cases each year, leading to tens of thousands of antibiotic courses being avoided [42]. In Italy, based on the results of a multi-cohort Markov model, vaccinating adults over 75 years old and high-risk patients with the adjuvanted RSVPre3OA vaccines would decrease LRTIs by 43.1% and antibiotic prescriptions by 43.2%, assuming a vaccine coverage of 75% [45]. Overall, incorporating RSV vaccines into routine immunizations, such as for infants via maternal immunization and for the elderly and vulnerable patients, seems a promising approach to decrease antibiotic use (sometime misuse) caused by RSV infection and complications.

### 3.5. Rotavirus Vaccine

In principle, rotavirus vaccination might significantly decrease antibiotic use by preventing diarrheal diseases that could sometimes be improperly treated with antibiotics. In HIC pediatric populations, widespread infant rotavirus immunization has been linked to fewer antibiotic prescriptions for gastroenteritis. A U.S. cohort study including over 2.1 million children found that those who completed a full rotavirus vaccination series had a 21% (95% CI 17–24%) lower risk of receiving antibiotics for acute gastroenteritis by age 5. Overall, this resulted in an estimated 67,045 (95% CI 52,729–80,664) avoided antibiotic prescriptions. These reductions occur because rotavirus is a leading cause of pediatric diarrhea and often prompts unnecessary antibiotic treatment; vaccines prevent these infections and thus decrease needless antibiotic use [46]. There is a limited direct impact on adults in HICs, who rarely need antibiotics for viral diarrhea. However, herd immunity from vaccinating infants could have also reduced rotavirus infections in older age groups.

In Italy, a retrospective analysis was conducted using hospital discharge records and vaccination coverage data for children aged 0–71 months from 2009 to 2019, to assess trends in standardized hospital discharge incidence before and after the introduction of the vaccine [47]. Vaccination coverage steadily increased over the years, rising from less than 5% between 2009 and 2013 to 26% in 2017, and reaching 70% by 2019. During the same period, the standardized incidence of hospital discharges decreased from 16.6 per 100,000 inhabitants in 2009–2013 to 9.9 per 100,000 inhabitants in 2018–2019. It is estimated that approximately 15% of hospital discharges were prevented [47].

### 3.6. Meningococcus B (MenB)

Vaccination against *Neisseria meningitidis* serogroup B has reduced the incidence of invasive meningococcal disease (IMD) in children, consequently decreasing the need for antibiotic treatments.

The UK’s national infant MenB immunization program, introduced in 2015, resulted in a 62% decline in MenB cases among vaccine-eligible young children within three years. An estimated 277 cases of meningococcal B meningitis/sepsis were prevented in the UK between 2015 and 2018 due to this program. Since virtually all IMD cases would have required hospitalization and intravenous antibiotics, preventing 277 (95% CI 236–323) cases equates to at least 277 antibiotic treatment courses avoided, along with a reduction in post-exposure antibiotic prophylaxis for numerous close contacts that each case would have required [48].

In adolescents and young adults, MenB vaccination campaigns have similarly reduced disease clusters. For example, targeted MenB vaccination at a U.S. university during an outbreak stopped transmission, preventing further cases and the use of prophylactic antibiotics in the student community [49].

In Italy, Men B is the prevalent serogroup in all age groups [50]. The increasing vaccination coverage rate in the pediatric age achieved in recent years with the 4CMenB vaccine has allowed a significant decrease in the incidence of IMD in children due to the high vaccine effectiveness [51]. Noteworthy, in a context where the incidence of IMD is limited and the impact of vaccination is high, the prescriptions of high doses of third-generation cephalosporins combined with aminoglycosides or vancomycin are suggested for a duration of 7–14 days in all suspected cases; this could contribute to the high level of resistance to third-generation cephalosporins registered in Italy [10].

It should be noted that MenB vaccines have shown some cross-protection against *Neisseria gonorrhoeae*, presenting an interesting future benefit in the battle against AMR. The cross-protection may be attributed to the high genetic and antigenic homology (80–90%) between *N. meningitidis* and *N. gonorrhoeae* [52]. Declining gonococcal cephalosporin susceptibility has raised the possibility of untreatable gonorrhea in the future. New approaches, such as vaccination, are needed as long-term strategies for gonorrhea prevention and control. In a recent systematic review and meta-analysis out of eight papers resulted that two doses of MenB-4C vaccine were 33% to 40% effective against gonorrhea [52]. In UK a world first 4CMenB for gonorrhea (further to meningitis) selective vaccination program primarily targeting gay, bisexual and other men who have sex with men in specialist sexual health services was introduced in September 2025 [53]. In Italy in 2023 the total number of sexually transmitted disease (STI) reports continues to rise, with an increase of 16.1% compared to 2021. The most significant increases observed in 2023 compared to 2021 were for gonorrhea (+83.2%), primary/secondary syphilis (+25.5%), and chlamydia infection (+21.4%) [54]. According to ECDC, in 2022 in Italy, 22% of gonococcal isolates were resistant to azithromycin and 84% to ciprofloxacin [55]. No immunization programs for STIs/gonorrhea are currently recommended in Italy.

### 3.7. Meningococcus ACWY (MenACWY)

In HICs, quadrivalent meningococcal conjugate vaccines, which target serogroups A, C, W, and Y, are typically given during childhood and adolescence to protect against meningitis and to build herd immunity. In a systematic review and metanalysis the effectiveness of meningococcal C vaccines for group C IMD (odds ratio [OR], 0.13 [95% confidence interval {CI}, 0.07–0.23]), and meningococcal A, C, W, Y (MenACWY) vaccines against group ACWY IMD (OR, 0.31 [95% CI, 0.20–0.49]) was confirmed [56].

The UK’s adolescent MenACWY campaign, launched in August 2015 to control a hyper-virulent MenW strain outbreak, quickly controlled the epidemic. Within a year, MenW cases stabilized and then sharply declined. Among 14- to 18-year-olds, the incidence of MenW dropped to an incidence rate ratio (IRR) of 0.35 (95% CI 0.17–0.76), and MenY to an IRR of 0.21 (95% CI 0.07–0.59), after the vaccination program was introduced. Intriguingly, modeling suggests that most cases were averted through herd effects, with between 205 and 1193 MenW cases prevented in the wider community, including unvaccinated groups, during the initial years, compared to approximately 25 cases prevented directly through vaccination. Similarly, for MenY, an estimated 60–106 cases were indirectly prevented for 19 cases directly protected. This highlights that vaccinating adolescents not only safeguards younger children and older adults from infection but also prevents hundreds of meningitis cases and the antibiotic treatments and prophylactic measures that would otherwise be necessary [57]. Adult populations in HICs benefit from these herd effects. However, routine ACWY vaccination is not offered to the general adult population.

In Italy, serogroup Y and W were the most prevalent after Men B among age classes ranging from 10 to >64 years [50].

### 3.8. Pertussis

Specific data on the impact of pertussis vaccination on antibiotic consumption were not retrieved from the search. Pertussis is very severe among newborns and most of the cases were reported in children below 5 years of age [58]. WHO Guidelines on pertussis considered antibiotic therapy not impactful on the clinical course but possibly reducing contagiousness [59]. A retrospective cohort study recently carried out in Italy on 148 pertussis cases showed that early treatment with macrolides for infants with pertussis improved clinical symptoms [60].

Pertussis is also very contagious among adults and is transmitted by air. The symptoms of the illness mainly affect the airways and, in the first weeks, are superimposable to those of influenza, such as catarrh, fever, fatigue, and conjunctivitis, and complications can occur, especially with advancing age, including pneumonia, sinusitis, and urinary incontinence [61]. Such a clinical presentation, especially in the winter period, may lead to antibiotic prescriptions as an empiric therapy in the community setting [61].

In Italy in 2019, pertussis incidence was 1.3/100.000 among the general population [58] but a recent evaluation in 2019 depicted a rate of pertussis underestimation by a factor of 4041 (in subjects older than 15 years) [62].

### 3.9. Measles

Specific data on the impact of measles (mumps, rubella) vaccination on antibiotic consumption were not retrieved from the search. In Italy, during the year 2017, 4991 cases of measles were recorded in Italy, which gave rise to complications in 35.8% of cases. Examining only superinfections, there were 378 cases of pneumonia and 225 cases of otitis that certainly required antibiotic therapy [63]. More recently, a resurgence of measles was observed in many countries, including Italy, in the time period of August 2023 to January 2025; 1164 cases (1065/91.5% laboratory confirmed) were reported overall and nearly 90% of them were unvaccinated [63]. Among the measles cases, a total of 403 cases (34.6%) reported at least one complication, including diarrhea (*n* = 131; 11.3%), pneumonia (*n* = 125; 10.7%), keratoconjunctivitis (*n* = 95, 8.2%), and otitis media (*n* = 29; 2.5%). Overall, 585 of 1156 cases (50.6%) for whom information is available required hospital admission, and 211 cases (18.3%) received outpatient care in a hospital emergency department [64].

### 3.10. Herpes Zoster

No specific data on the impact of herpes zoster vaccination on antibiotic consumption has been retrieved from the search. Additionally, the clinical impact of herpes zoster is well documented, as well as its high incidence of complications. Among these, bacterial superinfections have been reported, and are possibly related to inappropriate prescriptions of antibiotics [65].

## 4. Discussion

Operating according to the WHO Action Framework recommendations, this narrative review is intended to increase knowledge and awareness among Italian healthcare workers, professional medical associations, academic researchers, and public health advocates on the positive impact of vaccinations on AR, and also to broaden the perception of the value of vaccinations and increase vaccine confidence and acceptance.

In Italy, AMR, mainly AR, is an urgent health issue. The One Health strategy was included by the Ministry of Health within the AMR containment strategy [17]. Furthermore, the PNCAR and the PNPV plans were connected (as recommended by WHO), and many scientific societies have endorsed vaccination as a strategic pillar in tackling AMR [17,18,19]. Thus, the institutional and scientific frame is completed; however, different gaps must still be addressed before vaccines are considered as a tool to counteract AMR by the vast majority of healthcare providers (HCPs).

The literature search provided evidence on the potential impact that the immunizations included in the Italian PNPV might exert in tackling AR; for other vaccinations, the impact was postulated on the basis of the available data (Table 1). Influenza and pneumococcal vaccinations proved to be those with the broadest base of evidence for reducing antibiotic prescriptions. During the flu season of 2024–2025 in Italy, influenza-like syndromes affected 27.7% of Italians (25.3% during the last season), for a total of approximately 16,138,000 cases since the beginning of the season. Characterization of a sample of 59.674 clinical samples gave 23% positive for influenza [66]. Official data for pneumococcal infections are available only for invasive diseases, which are of course a narrow part of pneumococcal infections [50]; however, it can be argued that pneumococcal infections across all ages play a significant role in the global burden of infective episodes and the consequent antibiotic prescriptions. Thus, increasing influenza and pneumococcal vaccination coverage would certainly contribute to consistently reducing antibiotic prescriptions and thus tackling AR. Preliminary local evidence also suggests a notable impact on reducing antibiotic use from RSV immunization among adults and older adults [45].

Rotavirus vaccination proved to reduce antibiotic prescriptions, while varicella disease was associated with relevant use of antibiotics [38,39,46]. Even though no data are available for the Italian context, as these two viral vaccinations are included in the Italian PNPV, their contribution in counteracting AR is to be postulated, even if not measured.

For the other PNPV immunizations considered in the present review, such as meningococcal, pertussis, measles, and herpes zoster vaccinations, no evidence was retrieved on any measured impact they may exert in reducing antibiotic use. Such a limitation in data availability may be due to the reduced rates of infections (such as meningococcal infections), no reported data on the clinical management with antibiotics (such as measles or herpes zoster) or to a lack of clinical diagnosis and huge underreporting in the surveillance system (such as pertussis). Nevertheless, further to the WHO recommendations [12], it should be intuitive for a healthcare provider that reducing the rates of infections overall would generate a reduction in antibiotic use as well as a further gain in health.

Even though existing studies have robustly demonstrated reductions in antibiotic prescriptions with select vaccines, methodological inconsistencies in measuring AR-related endpoints remain to be considered. Most studies rely on observational or cohort-based designs, introducing confounders. The link between reduced antibiotic use and AR outcomes is often indirect rather than demonstrated causally. To strengthen the evidence base, controlled trials assessing the quantitative reduction in AR attributable to vaccines are needed [67]. However, such a goal is difficult to reach.

Limited real-world evidence and model gaps hinder an accurate assessment of vaccines’ impact on antibiotic use and AMR. There is a need to integrate AMR endpoints into vaccination programs and improve cost-effectiveness models to reflect long-term benefits and evolving AMR dynamics. On the other side, due to methodological difficulties, HCPs may, in the short term, trust the institutional recommendations and suspend their expectation in terms of stringency of evidence. In this perspective, it might be useful to keep in mind the so called “parachute paradox”, where no evidence was found according to Evidence-Based Medicine methods of the effect of a parachute in reducing mortality compared to not wearing a parachute when jumping out of an airplane [68,69].

In Italy, as in other countries, AMR management and prevention encompasses different scientific specialties and networks, such as infectious disease specialists, public health/preventive medicine specialists, HCPs, general practitioners, pediatricians, etc. Therefore, interdisciplinary teams should be arranged to effectively advocate for the value of vaccines in tackling AMR, mainly when managing comorbid patients. As an example, oncologists should also be engaged in infection management to tackle the issue of AMR, as the outcomes of patients with cancer worsen dramatically if MDR microorganisms are responsible for infections [70].

To more effectively engage different stakeholders, tailored messaging should be shaped according to different targets. As proposed by some UK experts, for the vaccine community it could be effective to highlight that vaccines not only prevent death and lessen disease severity but also help reduce AMR; for the AMR community, it should be emphasized that vaccines represent a crucial intervention alongside other strategies to combat AMR [67].

A still diffuse perception in Italy is that bacterial vaccines play a direct role in tackling AR, while vaccines for viral infections (e.g., influenza, RSV, and COVID-19) are quite less relevant. A survey carried out in Italy in 2020 among 51 experts documented that pertussis and meningococcal vaccinations were considered much more effective in counteracting AR compared to measles and varicella [71].

The present study is affected by some limitations. The literature search was carried out in only one database (Pubmed). Even though a fast check in the Embase database with the same search strategy did not significantly modify the Pubmed search results, it cannot be excluded that some papers with relevant results for the review might have been missed.

The data selection was conducted from a one country perspective, focusing on the vaccinations included in the Italian immunization calendar to summarize evidence instrumental for the Italian scientific community.

## 5. Conclusions

In conclusion, the role of immunization in reducing AMR has long been underestimated, but vaccines, given their direct and indirect effects, are essential in the fight against AMR. In this review the evidence on the impact that the vaccinations included in the Italian Immunization Plan may exert was compacted. The impact of vaccines in reducing AMR should be recognized by Italian stakeholders from both AMR and immunization communities, and strategies and implementation plans should always include vaccines as interventions to reduce AMR, supporting their widespread use.

There is no time left, the Italian scientific community is called to increase the focus on promoting vaccine uptake as a primary strategy for addressing AMR ahead of alternative approaches.

## Figures and Tables

**Table 1:** Type of evidence for the impact of selected vaccinations included into the Italian national immunization plan on the reduction in use of antibiotics.

Documented	Postulated
Pneumococcus	Pertussis
Influenza	Meningococcus B
Rotavirus	Meningococcus ACWY
Varicella	Measles
Respiratory Syncytial Virus	Herpes Zoster

## Data Availability

The data summarized in this review are from published articles and are publicly available.
